# Dyslipidemia Treatment in Patients with Acute Coronary Syndrome: Is It Time to Move to Combination Therapy?

**DOI:** 10.3390/jcm14186445

**Published:** 2025-09-12

**Authors:** Daniel Miron Brie, Cristian Mornoș, Ovidiu Adam, Alexandru Tîrziu, Alina Diduța Brie

**Affiliations:** 1 Cardiovascular Disease Institute Timisoara, Gheorghe Adam St., No. 13A, 300310 Timișoara, Romaniamornos.cristian@umft.ro (C.M.); 2 Research Center of the Institute of Cardiovascular Diseases, Gheorghe Adam St., No. 13A, 300310 Timișoara, Romania; 3 Department of Cardiology, “Victor Babes” University of Medicine and Pharmacy Timisoara, Eftimie Murgu Square, No. 2, 300041 Timișoara, Romania; 4 Department of Pediatric Surgery and Orthopedics, “Victor Babes” University of Medicine and Pharmacy, Eftimie Murgu Square, No. 2, 300041 Timișoara, Romania; 5 Department of Functional Sciences, “Victor Babes” University of Medicine and Pharmacy, Tudor Vladimirescu Street, No. 14, 300174 Timișoara, Romania; 6 ANAPATMOL Research Center, “Victor Babes” University of Medicine and Pharmacy, Tudor Vladimirescu Street, No. 14, 300174 Timișoara, Romania; alina.brie@umft.ro; 7 “Louis Țurcanu” Emergency Children Hospital, Doctor Iosif Nemoianu Street, No. 2, 300011 Timișoara, Romania

**Keywords:** acute coronary syndrome, dyslipidemia, lipid-lowering therapy, statins, ezetimibe, PCSK9 inhibitors

## Abstract

Dyslipidemia is a major modifiable risk factor in patients with acute coronary syndrome (ACS), and effective management is essential to reduce the risk of recurrent cardiovascular events. Recent guidelines emphasize early, intensive lipid-lowering therapy (LLT) and increasingly recommend combination regimens to achieve ambitious low-density lipoprotein cholesterol (LDL-C) targets. This review evaluates current evidence and recommendations for dyslipidemia treatment in ACS, with a focus on the rationale, timing, and selection of combination therapy. We conducted a comprehensive review of recent clinical guidelines, randomized controlled trials, and observational studies addressing lipid management in ACS. The analysis included data on LDL-C targets, efficacy and safety of high-intensity statins, adjunctive non-statin therapies (ezetimibe, PCSK9 inhibitors), and the impact of dietary interventions. Early and intensive LLT, initiated within 24–48 h of ACS, is associated with significant reductions in recurrent events and mortality. High-intensity statins (atorvastatin 40–80 mg or rosuvastatin 20–40 mg) are first-line, with combination therapy (statin plus ezetimibe and/or PCSK9 inhibitor) recommended for patients not achieving LDL-C < 1.4 mmol/L (<55 mg/dL) or >50% reduction from baseline. Evidence supports further LDL-C lowering (<1.0 mmol/L) in very high-risk patients. The Mediterranean and DASH diets provide additional benefit in lipid profile optimization and risk reduction. Statins also confer pleiotropic effects, including anti-inflammatory and plaque-stabilizing actions. Recent studies and real-world data confirm the efficacy and safety of combination approaches but highlight the need for individualized therapy based on residual risk, comorbidities, and tolerability. Achieving guideline-recommended LDL-C targets in ACS patients often requires early initiation of combination lipid-lowering therapy. Optimal management should be individualized considering both LDL-C levels and broader risk profiles. Ongoing research is needed to refine patient selection for combination therapy and to integrate novel agents into clinical practice.

## 1. Introduction

**Figure 1:**
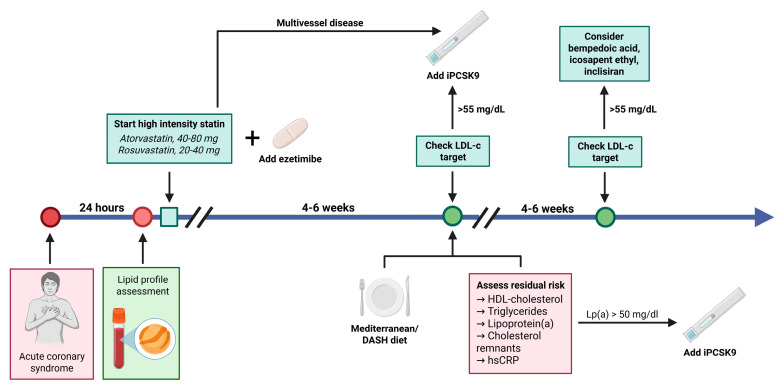
Current trends in lipid-lowering therapy after an acute coronary syndrome. A potent combination (atorvastatin or rosuvastatin with ezetimibe, 10 mg) is used and LDL-C levels are reevaluated after 4–6 weeks. If the LDL-C target is not achieved (LDL-C > 55 mg/dL), PCSK9 inhibitors should be added. Additionally, the residual risk must be assessed using HDL-cholesterol, triglycerides, lipoprotein (a), cholesterol remnants, hsCRP, and a Mediterranean/DASH diet should be adopted. In case residual risk persists, PCSK9 inhibitors should be added to the lipid-lowering therapy. At the second reassessment, if the LDL-cholesterol levels are still elevated, other options such as bempedoic acid, icosapent ethyl, or inclisiran can be considered. In case of multivessel disease diagnosed using coronary angiography during the index procedure, it is recommended to start with triple lipid lowering therapy (statin plus ezetimibe plus PCSK9i). In case of MINOCA, stepwise lipid lowering therapy should be initiated, as recommended by the ESC guidelines. Created in BioRender. Tirziu, A. (2025), https://BioRender.com/f2r71i0.

Dyslipidemia is a significant risk factor for acute coronary syndrome (ACS), and its management is crucial for reducing the risk of recurrent cardiovascular events. Recent research emphasizes the importance of early and intensive lipid-lowering therapy (LLT) in achieving optimal low-density lipoprotein cholesterol (LDL-C) levels and enhancing clinical outcomes. This paper provides a comprehensive review of treatment recommendations for dyslipidemia in patients with ACS based on the most relevant studies (Figure 1). 

According to the 2023 European Society of Cardiology (ESC) guidelines for the management of dyslipidemia [1] (in conjunction with the 2020 ESC guidelines on ACS [2], the treatment of dyslipidemia in patients with ACS focuses on achieving rapid and intensive LDL-C reduction to lower the risk of recurrent cardiovascular events. According to the current ESC guidelines, a lipid profile should be obtained as early as possible after admission for an ACS event, ideally within the first 24 h. This early measurement (even if non-fasting) best reflects the patient’s baseline lipid status, as lipid levels can change rapidly following ACS onset [3,4]. Lipid levels measured in the first 24 h provide a reliable baseline for assessing the patient’s usual lipid status, as mean lipid levels vary relatively little during the initial days after ACS [4].

According to the current guidelines of ESC, the recommended targets for LDL-C in patients with ACS are to reduce LDL-C levels to less than 1.4 mmol/L (<55 mg/dL) and by at least 50% from baseline levels. For patients who experience a second vascular event within two years while on maximally tolerated lipid-lowering therapy, a further reduced target of LDL-C < 1.0 mmol/L (<40 mg/dL) is advised.

The ambitious targets underscore the significant cardiovascular risk present in patients with ACS and are supported by robust evidence indicating that lower levels of LDL-C are correlated with a decreased incidence of recurrent cardiovascular events.

The risk of recurrence after an ACS is high, with an approximate rate of 6.7% (95% CI, 6.3–7.1%) at one year and 18.4% (95% CI, 17.4–19.5%) at nine years, as reported in a study from France [5]. The 5-year event rate for major cardiovascular events (nonfatal MI, nonfatal stroke, or cardiovascular death) is approximately 33.4%.

The risk of recurrence is highest immediately after discharge, with an annualized risk of approximately 40.9% in the early post-ACS period, decreasing to around 6.4% per year after the first year [6]. In specific cohorts, the 1-year recurrence rate ranges from about 4.2% in patients without previous MI to 11.9% in those with a prior MI [7]. Other studies report a 1-year recurrence rate of 12.35%, and the 1-year composite rate of cardiovascular death, recurrent MI, and stroke can be as high as 18.3% [8].

In conclusion, the risk of recurrent ACS or major cardiovascular events after an initial ACS is substantial, particularly within the first year, and remains elevated in the following years.

Lower LDL-C levels are strongly associated with a reduced risk of recurrent cardiovascular events after an ACS. Achieving lower LDL-C targets through intensive lipid-lowering therapy significantly decreases the likelihood of subsequent ACS events and overall cardiovascular morbidity and mortality. In one study, the adjusted hazard ratio for recurrent events in patients with LDL-C <70 mg/dL was 0.29, indicating a substantial risk reduction [9]. For each 1 mmol/L reduction in LDL-C, all-cause mortality declines by 10%, and the number of recurrent myocardial infarction events is reduced by 13% [10].

To achieve the proposed LDL-C cholesterol targets, the ESC guideline for ACS management [2] recommends a stepwise evaluation. Initially, the patient receives a potent statin (atorvastatin or rosuvastatin) at the maximum tolerated dose, followed by an assessment at 4–6 weeks. If the targets have not been reached (a reduction of at least 50% from baseline or an LDL-C lower than 1.4 mmol/L [<55 mg/dL]), ezetimibe is added to the potent statin. The patient is reevaluated after another 4–6 weeks, and if the targets are still not reached, a PCSK9 inhibitor (alirocumab or evolocumab) is administered. Is this approach correct? Is it suitable for all patients? How do we select those who would benefit from earlier combination therapy? What factors should be taken into account? Do only LDL-C values matter? How do we accurately assess residual risk after an ACS? What is the role of the new lipid-lowering therapies? Through the review below, we aim to address these questions.

## 2. Impact of Specific Dietary Patterns and Interventions on Dyslipidemia Management in Acute Coronary Syndrome Patients

Dietary management is a cornerstone of secondary prevention after ACS, aiming to improve lipid profiles and reduce recurrent cardiovascular risk. Among various nutritional approaches, the Mediterranean diet and the DASH (Dietary Approaches to Stop Hypertension) diet have the most substantial evidence and guideline recommendations for managing dyslipidemia in ACS patients. The Mediterranean diet emphasizes high intake of fruits, vegetables, whole grains, legumes, nuts, and olive oil (rich in monounsaturated fats), moderate consumption of fish and poultry, low intake of red meat and processed foods, and moderate wine consumption. For ACS patients with dyslipidemia, adopting a Mediterranean dietary pattern is strongly recommended due to its robust evidence in improving lipid profiles and reducing recurrent cardiovascular events. The DASH diet is also effective, particularly when hypertension coexists. Both diets emphasize whole, minimally processed plant-based foods and healthy fats, which together support lipid management and overall cardiovascular health. The Mediterranean diet is rich in monounsaturated fatty acids (MUFAs), primarily from olive oil and nuts, which raise HDL cholesterol levels, enhancing reverse cholesterol transport [11]. High intake of fruits, vegetables, whole grains, legumes, and fish provides antioxidants, fiber, and omega-3 fatty acids that reduce LDL cholesterol and triglycerides by improving lipid metabolism and reducing oxidative stress [12]. Polyphenols and other bioactive compounds in Mediterranean foods reduce vascular inflammation and the oxidative modification of LDL particles, thereby further improving the quality of the lipid profile and reducing the risk of atherosclerosis [13]. The DASH diet emphasizes fruits, vegetables, whole grains, and low-fat dairy and limits saturated fat and cholesterol, leading to significant reductions in LDL and triglycerides. Higher adherence to the DASH diet correlates with lower LDL/HDL ratio and better overall lipid balance. Although not a direct lipid effect, improved blood pressure and insulin sensitivity reduce metabolic stress on lipid metabolism, indirectly benefiting lipid profiles. Both diets improve lipid profiles by reducing atherogenic lipoproteins (LDL, triglycerides) and improving protective HDL levels or ratios through nutrient-rich, low saturated fat, and high fiber food patterns. These changes contribute to reduced cardiovascular risk in ACS patients.

## 3. Which Statins Do We Use and in What Doses After an ACS?

After an ACS, current guidelines recommend initiating high-intensity statin therapy as soon as possible—ideally within the first 24–48 h of admission. The primary statins and their recommended doses are:

- Atorvastatin: 40–80 mg daily;
- Rosuvastatin: 20–40 mg daily.

These doses are chosen to achieve rapid and substantial LDL-C reduction, which is associated with improved cardiovascular outcomes after ACS. High-dose statin loading before percutaneous coronary intervention (PCI) has also been shown to reduce short-term adverse events, particularly in statin-naive patients. Other statins (such as simvastatin or pravastatin) have lower LDL-C–lowering potency but are not considered first-line for high-intensity therapy in the ACS setting. If LDL-C targets are not achieved with high-intensity statin alone, combination therapy with ezetimibe is recommended.

Statins competitively inhibit the enzyme HMG-CoA reductase, which is the rate-limiting step in the hepatic cholesterol biosynthesis pathway. This inhibition reduces the liver’s production of cholesterol [14]. As hepatic cholesterol synthesis decreases, the liver increases the expression of LDL receptors on hepatocyte surfaces. This enhances the clearance of low-density lipoprotein cholesterol (LDL-C) from the bloodstream, further lowering plasma LDL-C levels [15].

Statins provide cardiovascular benefits that extend beyond their LDL-cholesterol-lowering action. These effects are known as pleiotropic and involve increasing the synthesis of nitric oxide, enhancing endothelial function and vascular responsiveness, and reducing blood pressure and vascular inflammation [16]. Statins contribute to the stabilization of atherosclerotic plaques by mitigating vascular inflammation and oxidative stress, thereby reducing the likelihood of plaque rupture and subsequent acute events [17]. These agents lower inflammatory markers such as C-reactive protein, diminish the activity and number of inflammatory cells, and inhibit the synthesis of pro-inflammatory cytokines. Furthermore, statins impede the release of free radicals and decrease the oxidation of LDL-C, thereby alleviating oxidative stress within the vascular wall. Statins also reduce platelet aggregation and adhesion, lower fibrinogen levels, and decrease blood viscosity, collectively diminishing the risk of thrombosis. Additionally, statins inhibit the proliferation of vascular smooth muscle cells, which enhances plaque stability and reduces the risk of restenosis [17,18].

Isoprenoids play a central role in mediating the pleiotropic (cholesterol-independent) effects of statins. Statins inhibit HMG-CoA reductase, which not only reduces cholesterol synthesis but also decreases the production of isoprenoid intermediates such as farnesylpyrophosphate (FPP) and geranylgeranylpyrophosphate (GGPP). These isoprenoids are essential for the post-translational modification (isoprenylation) of small GTP-binding proteins, including Rho, Ras, and Rac [19].

The MIRACL (Myocardial Ischemia Reduction with Aggressive Cholesterol Lowering) study evaluated the effects of initiating high-dose atorvastatin (80 mg daily) soon after an acute coronary syndrome (unstable angina or non–Q-wave MI). Atorvastatin 80 mg daily, started within 24–96 h of ACS, reduced the combined incidence of death, nonfatal MI, cardiac arrest, or recurrent symptomatic myocardial ischemia requiring emergency hospitalization at 16 weeks compared to placebo (14.8% vs. 17.4%; relative risk 0.84; 95% CI 0.70–1.00; *p* = 0.048) [20].

The reduction was primarily due to fewer episodes of symptomatic ischemia requiring rehospitalization (6.2% vs. 8.4%; RR, 0.74; *p* = 0.02) and fewer strokes (12 vs. 24 events; *p* = 0.045). Atorvastatin lowered mean LDL-C from 124 mg/dL to 72 mg/dL (3.2 to 1.9 mmol/L) but had minimal effect on HDL-C. High-dose atorvastatin significantly reduced inflammatory markers, such as CRP, supporting the value of early intensive statin therapy in ACS [21]. Interesting, baseline HDL-C, but not LDL-C, predicted short-term prognosis; the clinical benefit of atorvastatin was not directly related to the degree of LDL-C reduction, suggesting possible pleiotropic (non-lipid) effects [22].

The PROVE IT-TIMI 22 study compared intensive statin therapy (atorvastatin 80 mg daily) with standard therapy (pravastatin 40 mg daily) in 4162 patients who had experienced an acute coronary syndrome (ACS) within the previous 10 days. The median follow-up was 24 months. Intensive therapy with atorvastatin 80 mg significantly reduced the incidence of the primary composite endpoint (all-cause death, myocardial infarction, unstable angina requiring hospitalization, revascularization after 30 days, or stroke) compared to pravastatin 40 mg. The primary endpoint occurred in 22.4% of the atorvastatin group versus 26.3% of the pravastatin group (hazard ratio [HR] ≈ 0.84, *p* = 0.005) [23]. The benefit of intensive statin therapy was seen as early as 30 days (3.0% vs. 4.2%, HR 0.72, *p* = 0.046) and persisted in stable patients through the end of the study (9.6% vs. 13.1%, HR 0.72, *p* = 0.003) [24]. Atorvastatin also reduced the risk of recurrent ACS, need for revascularization, and stroke compared to pravastatin, supporting the use of high-intensity statins in this patient population.

High-dose rosuvastatin (20–40 mg daily) is highly effective at lowering LDL cholesterol in ACS patients, comparable to atorvastatin. Studies show that intensive rosuvastatin therapy can achieve substantial reductions in LDL-C and help a majority of patients reach the guideline-recommended target [24,25].

Multiple studies and meta-analyses have shown that high-intensity atorvastatin and rosuvastatin have comparable efficacy in reducing major adverse cardiovascular events (MACE) and all-cause mortality in patients with acute coronary syndrome (ACS) [26,27]. Both atorvastatin and rosuvastatin at high doses are excellent choices for secondary prevention in ACS, with no clinically meaningful difference in cardiovascular outcomes or mortality. Choice of statin can be individualized based on patient characteristics, comorbidities, potential side effects, and physician preference. Rosuvastatin may provide slightly greater LDL-C lowering but may carry a marginally higher risk of new-onset diabetes and cataract.

The Swedish RIKS-HIA registry (Register of Information and Knowledge about Swedish Heart Intensive Care Admissions) offers substantial real-world evidence regarding the efficacy of statin therapy in patients with acute coronary syndrome (ACS). The adjusted relative risk of mortality at one year for individuals receiving statins was 0.75 (95% CI: 0.63–0.89; *p* = 0.001), indicating a 25% reduction in relative risk compared to those not receiving statins. This survival advantage was evident even after controlling for confounding variables, with the mortality difference manifesting early, within the first year following the ACS event. Statin therapy was well tolerated in the acute context, with no heightened risk of adverse effects compared to patients with stable coronary artery disease. Discontinuation of statin therapy following hospital admission for ACS was linked to the poorest prognosis, highlighting the critical importance of maintaining or initiating statin therapy during hospitalization [28,29,30].

Several studies, including the A to Z trial (simvastatin) [31], PACT trial (pravastatin) [32], FLORIDA trial (fluvastatin) [33], and PRINCESS trial (cerivastatin) [34], have investigated different statins and their timing, yielding mixed results concerning the statistical significance of primary endpoints.

## 4. Adding Ezetimibe to Statin Therapy in Acute Coronary Syndrome

Ezetimibe acts at the brush border of the small intestine, where it selectively inhibits the absorption of cholesterol from the intestinal lumen into enterocytes. Its primary molecular target is the Niemann-Pick C1-Like 1 (NPC1L1) protein, which mediates the uptake of cholesterol into enterocyte [35].

The landmark IMPROVE-IT trial demonstrated that adding ezetimibe to statin therapy in patients with recent ACS led to a significant reduction in LDL cholesterol and improved cardiovascular outcomes compared to statin alone. Specifically, the combination reduced the risk of major adverse cardiovascular events, including nonfatal myocardial infarction and nonfatal stroke [36]. The IMPROVE-IT trial was a comprehensive, randomized, double-blind, placebo-controlled study that included 18,144 patients who were stabilized following acute coronary syndrome (ACS). These patients were randomly assigned to receive either ezetimibe 10 mg in combination with simvastatin 40 mg or simvastatin 40 mg alone, with a median follow-up duration of six years. The combination therapy reduced the incidence of the primary composite endpoint compared to simvastatin alone, confirming that further lowering of LDL-C beyond statin monotherapy improves cardiovascular outcomes in high-risk post-ACS patients. The benefit was consistent across subgroups, including patients with diabetes, prior stroke, and prior coronary artery bypass grafting, and was observed even in those with baseline LDL-C levels below 70 mg/dL. In patients with diabetes, the absolute reduction in the 7-year primary endpoint event rate was 5.5% (hazard ratio 0.85), with notable decreases in myocardial infarction (24%) and ischemic stroke (39%) [37].

IMPROVE-IT was the first trial to demonstrate that a non-statin agent (ezetimibe) added to statin therapy provides incremental clinical benefit in reducing cardiovascular risk after ACS. The results reinforced the “lower is better” hypothesis for LDL-C, demonstrating a benefit with LDL-C reduction even to very low levels, and established that LDL-C lowering in and of itself (not just statin use) reduces cardiovascular events. Real-world registry data, such as from the extensive Polish PL-ACS registry, showed that upfront combination therapy with statin and ezetimibe in ACS patients significantly reduced all-cause mortality at 1, 2, and 3 years compared to statin monotherapy (absolute risk reduction of 4.7% at 3 years; number needed to treat = 21) [38]. A recent meta-analysis has demonstrated that the early incorporation of ezetimibe into high-intensity statin therapy for patients with acute coronary syndrome (ACS) results in significantly reduced low-density lipoprotein (LDL) cholesterol levels at all evaluated time points—day 7, 1 month, 3 months, and 1 year—compared to statin therapy alone. The mean difference in LDL-C ranged from −19.55 mg/dL at 7 days to −16.90 mg/dL at 10–12 months. This combination therapy significantly decreased the risk of recurrent cardiovascular events following an ACS event, including all-cause mortality, ACS, and non-fatal stroke (OR 0.89, 95% CI: 0.83–0.96), non-fatal myocardial infarction (OR 0.86, 95% CI: 0.79–0.94), and ischemic stroke (OR 0.80, 95% CI: 0.68–0.94) [39].

## 5. PCSK9 Inhibitor Administration in ACS

PCSK9 inhibitors block the PCSK9 protein, preventing degradation of LDL receptors on liver cells, thereby increasing LDL receptor availability and enhancing clearance of LDL-C from the bloodstream.

PCSK9 levels rise significantly early after ACS, making early initiation of PCSK9 inhibitors beneficial. Studies such as EPIC-STEMI [40] and PACMAN-AMI [41] demonstrate that early use of PCSK9 inhibitors can rapidly lower LDL-C, promote plaque regression, and reduce short-term cardiovascular event risk. Beyond LDL-C lowering, PCSK9 inhibitors contribute to stabilization and regression of atherosclerotic plaques, reducing plaque vulnerability and subsequent ischemic events [42]. PCSK9 promotes inflammation by upregulating pro-inflammatory cytokines and activating the NF-κB pathway in macrophages and other immune cells. PCSK9 inhibitors reduce this activation, thereby decreasing expression of inflammatory mediators such as IL-6, TNF-α, and MCP-1 [43]. PCSK9 inhibitors reduce inflammation by suppressing pro-inflammatory cytokine production, inhibiting immune cell activation, decreasing monocyte recruitment, and promoting anti-inflammatory pathways, thereby stabilizing atherosclerotic plaques and lowering cardiovascular risk beyond lipid lowering alone [44].

The FOURIER trial evaluated the efficacy and safety of the PCSK9 inhibitor evolocumab in patients with established atherosclerotic cardiovascular disease who were already undergoing statin therapy. Evolocumab resulted in a 59% reduction in LDL cholesterol compared to placebo, lowering the median LDL-C from 92 mg/dL to 30 mg/dL at 48 weeks. Evolocumab significantly decreased the risk of the primary composite endpoint, which included cardiovascular death, myocardial infarction, stroke, hospitalization for unstable angina, or coronary revascularization, by 15% compared to placebo (hazard ratio 0.85; 95% CI, 0.79–0.92; *p*< 0.001). Furthermore, there was a 20% reduction in the risk of cardiovascular death, myocardial infarction, or stroke (hazard ratio 0.80; 95% CI, 0.73–0.88; *p* < 0.001). The primary endpoint occurred in 9.8% of patients on evolocumab versus 11.3% on placebo over a median follow-up of 2.2 years. Evolocumab reduced not only the first occurrence but also the total number of cardiovascular events, including myocardial infarction, stroke, and coronary revascularization [45]. The incidence of serious adverse events, such as muscle-related events, new-onset diabetes, hemorrhagic stroke, and neurocognitive events, did not exceed those observed with placebo and did not increase over time. Injection-site reactions were slightly more common with evolocumab (2.1% vs. 1.6%). In the FOURIER-OLE extension, long-term LDL-C lowering with evolocumab was associated with sustained low rates of adverse events and further reduced cardiovascular event risk, with up to 8.4 years of exposure [46].

The ODYSSEY OUTCOMES trial evaluated alirocumab, a PCSK9 inhibitor, in 18,924 patients with recent acute coronary syndrome (ACS) who were already on high-intensity or maximum tolerated statin therapy. Alirocumab significantly reduced the risk of major adverse cardiovascular events (MACE: coronary heart disease death, nonfatal myocardial infarction, fatal or nonfatal ischemic stroke, or unstable angina requiring hospitalization) compared to placebo (9.5% vs. 11.1%; hazard ratio 0.85, 95% CI 0.78–0.93; *p* < 0.001) [47]. Alirocumab reduced both first and total nonfatal cardiovascular events and deaths, with the total number of events prevented being about twice the number of first events prevented (hazard ratio for total events: 0.87, 95% CI 0.82–0.93) [48].

Early initiation of PCSK9 inhibitors in hospitalized ACS patients leads to rapid and significant reductions in LDL cholesterol and lipoprotein(a) compared to statin therapy alone or placebo. Meta-analyses show LDL-C reductions of up to 54% and Lp(a) reductions of about 27% when PCSK9 inhibitors are added to high-intensity statins in this setting [49]. Early PCSK9 inhibitor therapy significantly reduces the risk of major adverse cardiovascular events, including nonfatal myocardial infarction, cardiogenic death, stroke, hospitalization for recurrent ACS, and coronary revascularization. Pooled data from randomized controlled trials demonstrate a reduction in MACE with an odds ratio (OR) of 0.83 (95% CI: 0.77–0.88) compared to placebo. Most current guidelines recommend starting PCSK9 inhibitors in patients with acute coronary syndrome (ACS) if LDL-C targets are not achieved after 4–6 weeks of maximally tolerated statin and ezetimibe therapy. However, there is increasing evidence and expert discussion supporting earlier initiation, even during the acute hospital phase, especially in patients with very high LDL-C or high cardiovascular risk despite optimal oral lipid-lowering therapy [50,51]. Multiple recent studies and systematic reviews support the safety and efficacy of early, in-hospital initiation of PCSK9 inhibitors in patients with acute coronary syndrome (ACS). Early initiation of PCSK9 inhibitors, defined as administration during hospitalization or within four weeks following the onset of acute coronary syndrome (ACS), has been demonstrated to rapidly and significantly reduce levels of low-density lipoprotein cholesterol (LDL-C), triglycerides, and non-high-density lipoprotein cholesterol. This approach increases the proportion of patients achieving LDL-C targets before discharge. It decreases the incidence of major adverse cardiovascular events (MACE), including coronary revascularization, recurrent ACS, readmissions for unstable angina, and potentially stroke. Importantly, this strategy does not significantly elevate the occurrence of adverse drug events compared to standard therapy [49]. The VCU-AlirocRT (Virginia Commonwealth University Alirocumab Response Trial) was a randomized, double-blind, placebo-controlled pilot study evaluating the safety and efficacy of early alirocumab administration in patients with non-ST-elevation myocardial infarction (NSTEMI) who had LDL-C levels greater than 70 mg/dL despite high-intensity statin therapy. Alirocumab led to a dramatic reduction in LDL-C at 14 days: a median decrease of 64 mg/dL (interquartile range: −96 to −47) compared to a 1 mg/dL change (−25 to +16) with placebo. Significant LDL-C lowering was already evident by day 3, with the mean LDL-C reaching 28 mg/dL at 14 days in the alirocumab group [52].

The EVACS (Evolocumab in Acute Coronary Syndrome) trial investigated the safety, feasibility, and efficacy of initiating the PCSK9 inhibitor evolocumab during hospitalization for acute coronary syndrome (ACS), specifically targeting patients with non-ST-elevation myocardial infarction (NSTEMI). Evolocumab was administered within 24 h of hospital presentation for NSTEMI, in conjunction with standard statin therapy. Notably, two-thirds of patients who received evolocumab within this timeframe achieved LDL-C targets (<1.8 mmol/L, or ~70 mg/dL) and were discharged with LDL-C levels at goal. The in-hospital initiation of evolocumab is both safe and effective in rapidly reducing LDL-C in this high-risk population [50].

The EVOPACS (Evolocumab for Early Reduction of LDL Cholesterol Levels in Acute Coronary Syndrome) trial was a randomized, double-blind, placebo-controlled multicenter study involving 308 patients with new-onset acute coronary syndrome (ACS). It was the first trial to evaluate the lipid-lowering effectiveness of early initiation of the PCSK9 inhibitor evolocumab in ACS patients. Mean LDL-C dropped from 3.61 mmol/L (approximately 140 mg/dL) to 0.79 mmol/L (~30 mg/dL) in the evolocumab group after 8 weeks compared to a reduction from 3.42 mmol/L (132.3 mg/dL) to 2.06 mmol/L (79.6 mg/dL) in the placebo group. Over 95% of patients receiving evolocumab achieved guideline-recommended LDL-C targets (<1.4 mmol/L or ~55 mg/dL) by 8 weeks. Total cholesterol decreased by 26.5%, apolipoprotein B by 34.2%, and non-HDL cholesterol by 34.6% in the evolocumab group compared to placebo [53].

In patients with NSTEMI, it is sometimes difficult to identify the “culprit” lesion responsible for the acute symptoms, as most patients have multivessel disease with multiple atherosclerotic lesions. Some studies show that NSTEMI patients may have slightly higher long-term mortality compared to STEMI, especially due to non-cardiovascular causes, but after adjustment for confounders, the difference is often not significant [54]. Spadafora et al. found that STEMI and NSTEMI patients have similar one-year outcomes, with reinfarction risk differences stemming from baseline patient characteristics and treatment approaches rather than infarct classification [55].

The HUYGENS trial (High-Resolution Assessment of Coronary Plaques in a Global Evolocumab Randomized Study) investigated the effects of adding the PCSK9 inhibitor evolocumab to maximally tolerated statin therapy in patients with NSTEMI. Evolocumab significantly increased fibrous cap thickness compared to placebo (mean increase: 42.7 µm vs. 21.5 µm; *p* = 0.01), indicating improved plaque stability. There was a decrease in the maximum lipid arc and a reduction in macrophage infiltration, both of which are markers of plaque vulnerability. These findings suggest that early, intensive lipid lowering with evolocumab, on top of statin therapy, after ACS not only reduces LDL-C but also favorably alters high-risk plaque features, potentially reducing residual cardiovascular risk [56].

The PACMAN-AMI (Plaque Characterization with Alirocumab in the Global Assessment of Plaque Regression with a PCSK9 Antibody as Measured by Multimodality Imaging After Acute Myocardial Infarction) trial was a multicenter, randomized, double-blind, placebo-controlled study evaluating the effects of early alirocumab initiation on coronary plaque characteristics in patients with AMI. Patients were randomized to receive alirocumab (150 mg subcutaneously every two weeks) or placebo, starting within 24 h after PCI, on top of high-intensity statin therapy (rosuvastatin 20 mg). At 52 weeks, a more pronounced reduction in mean percent atheroma volume (PAV) was observed with alirocumab compared to placebo (−2.13% vs. −0.92%; *p* < 0.001). Additionally, there was a significant decrease in the total lipid core burden index (LCBI) as measured by NIRS (−29.3 vs. −12.4; *p* = 0.004) [57]. The study concluded that early and intensive lipid-lowering treatment with alirocumab, in conjunction with high-intensity statin therapy, results in more significant coronary plaque regression, lipid core reduction, and plaque stabilization in patients with acute myocardial infarction (AMI) compared to statin therapy alone. The trial provides substantial mechanistic evidence supporting the early administration of PCSK9 inhibitors following acute myocardial infarction (AMI) to reduce residual atherosclerotic risk. This is particularly applicable for patients with acute coronary syndrome (ACS) and multiple atherosclerotic coronary lesions, and to a lesser extent for those experiencing myocardial infarction with non-obstructive coronary artery (MINOCA), where significant atherosclerosis is absent.

## 6. Bempedoic Acid: Mechanism and Rationale for Use in Acute Coronary Syndrome

Bempedoic acid is an oral, once-daily ATP citrate lyase inhibitor that lowers LDL-C by inhibiting cholesterol synthesis in the liver, with minimal risk of muscle-related side effects compared to statins [58]. It is particularly useful for patients who are statin-intolerant or unable to achieve LDL-C goals with statins alone [59]. Previous large trials (CLEAR program) demonstrated bempedoic acid’s efficacy in lowering LDL-C and reducing cardiovascular events in high-risk patients but excluded those with recent ACS [60]. Newer studies, such as the ES-BempeDACS [61], directly evaluate the efficacy and safety of early intensive lipid-lowering therapy using a combination of high-potency statin, bempedoic acid, and ezetimibe versus standard care in post-AMI patients. The authors conclude that early administration of triple therapy (statin + ezetimibe + bempedoic acid) in patients with STEMI enables them to reach the target goals (LDL-C < 1.4 mmol/L = 55 mg/dL) within 2 months of starting treatment, much faster compared to the classic stepwise approach. Future trials will determine the role of bempedoic acid administration as a lipid-lowering therapy in patients with ACS (ex. CLEAR-ACS - NCT05263778).

## 7. Role of Inclisiran in Patients with Acute Coronary Syndrome

Inclisiran is a small interfering RNA (siRNA) therapy that lowers LDL-C by inhibiting hepatic production of PCSK9, thus enhancing LDL receptor recycling and clearance of LDL-C from the bloodstream [62].

The VICTORION-INCEPTION trial (NCT04873934) is a phase IIIb, randomized, open-label, multicenter study (presented at the National Lipid Association’s 2025 Annual Scientific Sessions; 29 May–1 June 2025; Miami, Florida) designed to evaluate the efficacy of inclisiran in patients with recent acute coronary syndrome (ACS) who exhibit low-density lipoprotein cholesterol (LDL-C) levels exceeding guideline-recommended targets despite receiving standard care. The trial seeks to determine whether the early initiation of inclisiran, in conjunction with standard lipid-lowering therapy (including maximally tolerated statins), enhances LDL-C control and goal attainment in this high-risk cohort. The addition of inclisiran to usual care resulted in a rapid and sustained LDL-C reduction of approximately 45.6%, compared to a 1.4% increase observed with usual care alone. The trial demonstrated that early administration of inclisiran post-ACS can significantly improve the attainment of guideline-recommended LDL-C targets, which is crucial for the secondary prevention of cardiovascular events. The VICTORION-INCEPTION trial provides strong evidence that early use of inclisiran after ACS, in addition to standard care, is effective and safe for achieving LDL-C targets in high-risk patients, supporting its early adoption in post-ACS lipid management strategies.

## 8. Role of Icosapent Ethyl in Acute Coronary Syndrome

Icosapent ethyl (IPE), a purified eicosapentaenoic acid (EPA) derivative, has a well-established role in reducing cardiovascular events in high-risk patients, including those with recent ACS, particularly when added to statin therapy. In a post hoc analysis of the REDUCE-IT trial, IPE (4 g/day) significantly reduced the risk of both first and total ischemic events in statin-treated patients with recent ACS (within 12 months) compared to placebo [63]. IPE was associated with a 37% reduction in the incidence of first primary endpoint events (composite of cardiovascular death, nonfatal MI, nonfatal stroke, coronary revascularization, or unstable angina) and a 36% reduction in total primary events in this subgroup. Over 5 years, the absolute risk reduction for first events was 9.3% (number needed to treat [NNT] = 11), which is notably higher than in patients with more remote ACS. The benefit of IPE was observed regardless of baseline LDL-C, including patients with LDL-C < 55 mg/dL, supporting its use even in those with optimally controlled LDL-C but elevated triglycerides [64]. IPE reduces triglyceride levels, which are linked to residual cardiovascular risk after ACS, but the magnitude of cardiovascular benefit exceeds what would be expected from triglyceride lowering alone. IPE has been shown in preclinical and smaller clinical studies to reduce inflammation, which plays a key role in atherosclerosis progression and plaque instability and may stabilize vulnerable atherosclerotic plaques, reducing the risk of rupture and subsequent ischemic event [65].

The 2022 NICE guidelines recommend IPE for ACS patients with fasting triglycerides ≥1.7 mmol/L (≥150 mg/dL) and LDL-C between 1.04 and 2.60 mmol/L (40–100 mg/dL), despite statin therapy, reflecting the REDUCE-IT evidence. Evidence supports starting IPE as soon as possible after an ACS event in eligible patients, alongside standard secondary prevention therapies. Ideal candidates are those with established cardiovascular disease (including recent ACS), on statins, with controlled LDL-C (41–100 mg/dL) and elevated triglycerides (135–499 mg/dL) [66].

Icosapent ethyl reduces ischemic events after ACS primarily through a combination of triglyceride-lowering and multiple pleiotropic effects including anti-inflammatory action, plaque stabilization, improved endothelial function, and antithrombotic properties. These mechanisms collectively contribute to significant reductions in first and recurrent ischemic cardiovascular events observed in the REDUCE-IT trial [67].

## 9. Role of ANGPTL3 and ApoC-III Inhibitors in Acute Coronary Syndrome (ACS)

ANGPTL3 (Angiopoietin-like protein 3) and apoC-III (apolipoprotein C-III) are key regulators of lipid metabolism that contribute to elevated levels of triglyceride-rich lipoproteins (TRLs) and their remnants, which are atherogenic and implicated in residual cardiovascular risk after ACS. Both ANGPTL3 and apoC-III inhibit lipoprotein lipase (LPL), the enzyme responsible for hydrolyzing triglycerides in TRLs, leading to increased circulating TRLs and remnant cholesterol, which promote atherosclerosis [68]. ANGPTL3 inhibitors also lower LDL cholesterol via mechanisms that may be independent of the LDL receptor, including reduced hepatic secretion of ApoB-containing lipoproteins and increased clearance of LDL precursors [69]. ANGPTL3 inhibitors also lower LDL cholesterol via mechanisms that may be independent of the LDL receptor, including reduced hepatic secretion of ApoB-containing lipoproteins and increased clearance of LDL precursors [70].

ApoC-III inhibits LPL and hepatic lipase, delays clearance of TRL remnants by interfering with their hepatic uptake, and increases VLDL production, all contributing to hypertriglyceridemia and remnant cholesterol elevation. ApoC-III promotes vascular inflammation and atherogenesis through multiple pathways, including the modulation of LDL binding to the arterial wall and the upregulation of proinflammatory mediator [68]. Antisense oligonucleotides and RNA interference therapies targeting apoC-III are under clinical development, showing substantial reductions in triglycerides and potential to reduce ASCVD risk [70].

## 10. Evaluating Residual Dyslipidemia Risk After Acute Coronary Syndrome (ACS)

Residual dyslipidemia refers to persistent lipid abnormalities in patients who have already received standard lipid-lowering therapy after ACS. Assessing this risk is crucial because residual dyslipidemia is linked to higher rates of recurrent cardiovascular events. The initial assessment should include measuring LDL-C, HDL-C, and triglycerides (TGs). It is also important to measure Lp(a) levels as early as possible after ACS. Despite statin therapy, many ACS patients do not reach guideline-recommended targets (e.g., LDL-C < 55 mg/dL), and a significant proportion have low HDL-C or high TG. In a large cohort, 67.75% of ACS patients after PCI had suboptimal LDL-C, 85.85% had low HDL-C, and 33.64% had high TG despite statin therapy. Low HDL-C in particular was associated with a 41% increase in major adverse cardiovascular and cerebrovascular events (MACCEs) and a 48% increase in revascularization rates [71].

Elevated Lp(a) is an independent risk factor for MACE and all-cause mortality in ACS patients, even after accounting for other lipid parameters and standard therapies. Meta-analyses and large cohort studies consistently show that higher Lp(a) levels are associated with increased risk of recurrent events, including death, stroke, non-fatal myocardial infarction, and need for revascularization [72]. Lp(a) measurement can improve risk stratification beyond traditional lipid panels, especially in patients with a family history of premature cardiovascular disease or those with recurrent events despite optimal LDL-C lowering. High Lp(a) levels may identify patients who will derive greater absolute benefit from therapies such as PCSK9 inhibitors, which lower both LDL-C and Lp(a) and have shown greater event reduction in patients with elevated Lp(a). Guidelines recommend measuring Lp(a) at least once in a lifetime, particularly in high-risk groups, to enhance cardiovascular risk assessment and management [73]. Lp(a) ≥30 mg/dL is a strong predictor of coronary artery disease (CAD) in women, while ≥50 mg/dL is predictive in both men and women [74]. The 50 mg/dL cut-off is appropriate for White and Hispanic populations, while 30 mg/dL may be more relevant for Black individuals [75].

Remnant cholesterol refers to the cholesterol content found in triglyceride-rich lipoproteins, specifically very-low-density lipoproteins (VLDLs), intermediate-density lipoproteins (IDLs), and chylomicron remnants [76]. Remnant cholesterol particles are rich in triglycerides and are considered highly atherogenic, meaning they contribute to the buildup of fatty deposits (plaques) in arteries. Research suggests that remnant cholesterol may be a stronger predictor of heart disease and stroke than LDL cholesterol, especially in people with normal total cholesterol levels. High levels of remnant cholesterol can promote inflammation, endothelial dysfunction, and thrombosis, all of which contribute to the development of atherosclerosis and related complications [77].

Remnant cholesterol can be quantified through two primary methods: (1) Calculation, which involves subtracting high-density lipoprotein (HDL) and low-density lipoprotein (LDL) cholesterol from total cholesterol (Remnant cholesterol = Total cholesterol − HDL − LDL); and (2) Direct measurement, which employs specialized laboratory techniques such as ultracentrifugation, nuclear magnetic resonance spectroscopy, or automated assays. Remnant cholesterol is increasingly recognized as a significant factor in the prognosis and residual risk of patients following ACS, even after optimal statin therapy and LDL cholesterol control. After ACS, many patients remain at risk for future cardiovascular events despite achieving target LDL cholesterol levels. Remnant cholesterol is a major contributor to this “residual risk,” particularly in statin-treated patients [16]. Multiple studies and meta-analyses show that high remnant cholesterol levels independently predict MACEs in patients with coronary heart disease, including those with recent ACS [78]. RC is a better predictor of recurrent events than LDL cholesterol when LDL is well controlled [79]. Higher RC levels after ACS are linked to increased all-cause mortality and heart failure readmissions, especially when RC exceeds 60 mg/dL. Measuring RC after ACS can improve risk stratification, helping identify patients at higher risk for recurrent events and mortality, even if traditional lipid targets are met and may guide more intensive or targeted therapies.

**Figure 2:**
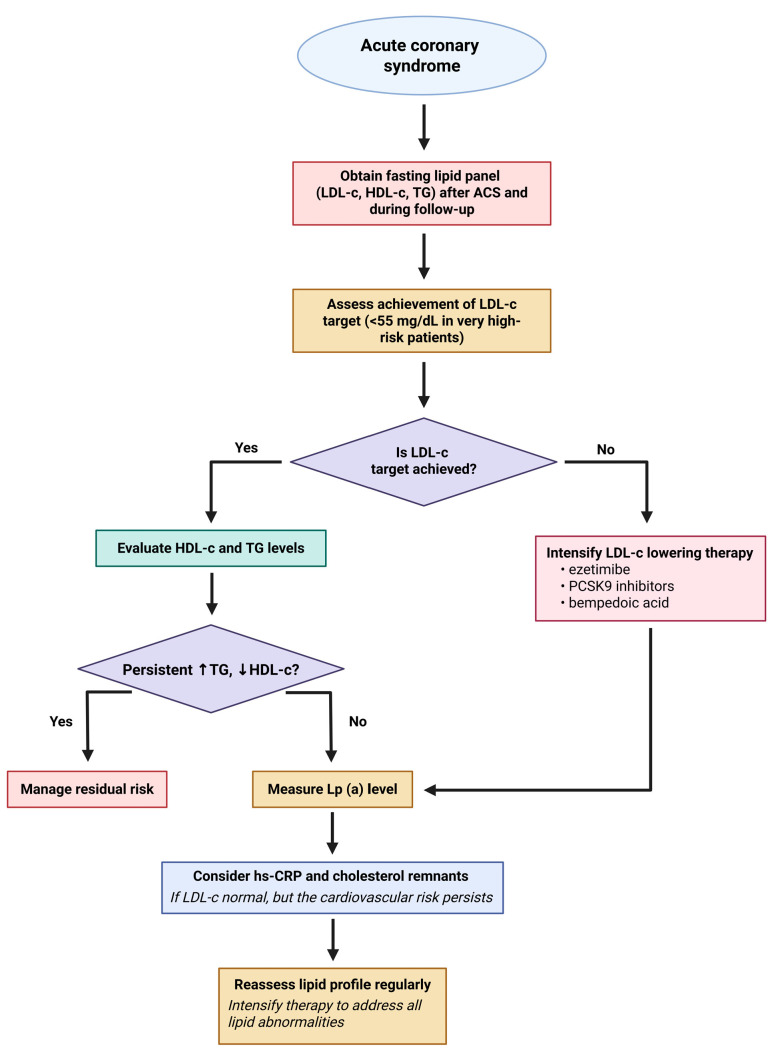
Flowchart outlining a stepwise approach to lipid management in patients following acute coronary syndrome. The algorithm begins with obtaining a fasting lipid panel and assessing whether the LDL-C target (<55 mg/dL in very-high-risk patients) is achieved. If not, LDL-C-lowering therapy should be intensified using agents such as ezetimibe, PCSK9 inhibitors, or bempedoic acid. If the target is achieved, HDL-C and triglyceride levels are evaluated. Persistent dyslipidemia (↑ TG, ↓ HDL-C) prompts management of residual risk. If not present, Lp(a) levels should be measured, and further evaluation with hs-CRP and cholesterol remnants considered. Regular reassessment and therapy intensification aim to address all lipid abnormalities to reduce cardiovascular risk. Created in BioRender. Tirziu, A. (2025) https://BioRender.com/npvpd7x.

Elevated remnant cholesterol and triglyceride-rich lipoproteins contribute to residual cardiovascular risk after ACS despite optimal LDL-C lowering. ANGPTL3 and apoC-III inhibitors represent novel therapeutic strategies targeting these atherogenic lipoproteins to further reduce ischemic events post-ACS. By enhancing TRL clearance, lowering triglycerides, remnant cholesterol, and LDL-C, while reducing vascular inflammation, these inhibitors may provide additional risk reduction in ACS patients with persistent dyslipidemia (Figure 2).

### 10.1. Discordance Between LDL-C and Other Atherogenic Markers

Although LDL-C, non-HDL-C, and ApoB are highly correlated statistically (Pearson’s R^2^ ≈ 0.96), discordance at the individual patient level is common and clinically meaningful. ApoB reflects the total number of atherogenic particles, while LDL-C and non-HDL-C indicate cholesterol content; patients with similar LDL-C or non-HDL-C levels can have widely varying ApoB values, leading to differences in the actual atherogenic risk [80]. Discordance analysis shows that elevated ApoB is associated with significantly increased CV risk—even at “controlled” LDL-C or non-HDL-C levels. Adjusted risk models find that when ApoB is high but LDL-C or non-HDL-C is low, CV event rates are higher. Conversely, patients with lower ApoB have reduced risk regardless of LDL-C [81]. This discordance is significant in statin users with low LDL-C but elevated ApoB or non-HDL-C [82] and also in individuals with diabetes, obesity, hypertriglyceridemia, or metabolic syndrome—where classic LDL-C underestimates risk [83]. Therefore, it is advised that routine assessments for patients with ACS include measurements of ApoB and/or non-HDL-C in addition to LDL-C. This is particularly important for individuals with mixed dyslipidemia, diabetes, obesity, or those who exhibit persistent risk despite statin therapy. In risk stratification, it is crucial to consider the discordance between LDL-C, ApoB, and non-HDL-C levels. In cases of discordance, ApoB should be prioritized as the most accurate marker for guiding therapeutic interventions.

### 10.2. ApoB and Non-HDL-C as Superior Predictors

Large meta-analyses and diverse cohort studies show ApoB and non-HDL-C are more robust predictors of ASCVD events than LDL-C, especially in statin-treated or complex profiles. ApoB is the direct measure of atherogenic particle number and captures risk that LDL-C may miss, such as cholesterol-depleted small LDL particles [83]. Recent lipid management guidelines increasingly recommend ApoB or non-HDL-C measurement, particularly as secondary targets in high-risk populations where LDL-C alone is insufficient to capture total risk. ApoB or non-HDL-C assessment is recommended for individuals with persistent residual risk, such as those with diabetes or persistent hypertriglyceridemia [84].

### 10.3. Risk Evaluation by Plaque Composition Beyond Lipid Values: Lipid Core Burden Index

Near-Infrared Spectroscopy (NIRS) plays a significant role in the evaluation of ACS by enabling the identification and quantification of lipid-rich plaques within the coronary arteries. This technology is available as a catheter-based modality, often combined with intravascular ultrasound (NIRS-IVUS), and it has been robustly validated against histopathology for its ability to detect fibroatheromas and lipid core plaques (LCPs) [85]. High lipid core burden index (LCBI), particularly maxLCBI 4 mm ≥ 400, as measured by NIRS—even in non-culprit artery segments—independently predicts MACE within follow-up periods [86]. Patients with a high LCBI in non-culprit arteries show up to a fourfold increased risk of adverse outcomes. NIRS can be used to stratify patients and specific arterial segments by risk, identifying those with vulnerable plaques most likely to result in future acute coronary events [87]. The technology is useful in both stable and unstable (ACS) patients, and its ability to detect plaque vulnerability is being actively evaluated in large, ongoing studies.

By identifying lipid-rich plaques, NIRS may help guide additional interventions, more intensive medical therapy, or closer surveillance in high-risk patients beyond what is predicted by coronary angiography or physiology alone. NIRS can identify regions at risk of plaque progression even before pronounced wall thickening, suggesting utility for early intervention and monitoring [88]. Integration of NIRS data with other imaging modalities (OCT, IVUS, CT) and computational techniques (machine learning, artificial intelligence) is employed to improve comprehensive risk stratification and predictive modeling for individual patients [89]. NIRS is currently explored as a tool for monitoring plaque progression or regression in response to targeted therapies such as PCSK9 inhibitors, novel anti-inflammatory agents, or other emerging drugs [90]. Longitudinal studies assessing whether changing the lipid core burden index (LCBI) over time due to therapies or lifestyle modification can be reliably linked to outcome improvements, providing new surrogate endpoints for clinical research [91].

The Lipid Core Burden Index (LCBI) is a quantitative measure that assesses the lipid (fatty) core content within an arterial plaque, utilizing imaging techniques such as Near-Infrared Spectroscopy (NIRS) or Optical Coherence Tomography (OCT). In Near-Infrared Spectroscopy (NIRS), the Lipid Core Burden Index (LCBI) is a quantitative measure of the amount of lipid-rich plaque within a selected region of interest in an artery [92]. The NIRS catheter scans the artery and generates a chemogram: a map where each pixel corresponds to a small segment of the arterial wall [93,94]. To each pixel a probability is assigned that lipid (cholesterol-rich) material is present, typically based on the NIRS spectral signature. Regions with higher probabilities (>0.6) to contain cholesterol-rich material are marked in yellow, while the other regions are marked in red. LCBI is a validated imaging biomarker for assessing the lipid content of coronary plaques, used to predict risk of cardiac events and inform clinical management of CAD patients, particularly in the context of PCI and research on atheroma reduction therapies.

The LCBI is calculated using the formula

$$LCBI=\frac{Number\;of\;yellow\;pixels\times 1000}{Total\;valid\;pixels}$$

Values range from 0 to 1000, indicating the proportion of lipid-rich areas in the examined vessel segment. The term maxLCBI 4 mm refers to the highest LCBI value recorded within any 4 mm segment of the scanned area [95].

LCBI is a key marker for identifying and quantifying high-risk plaques in coronary arteries, particularly those with large lipid cores that are prone to rupture. It is valuable for risk stratification in patients with coronary artery disease (CAD), both during and after percutaneous coronary intervention (PCI). From a management and prognostic perspective, plaques with elevated LCBI may require closer monitoring or more aggressive treatment. Additionally, LCBI serves as a surrogate marker in clinical research to assess the effectiveness of anti-atherosclerotic therapies.

Typical clinical thresholds include maxLCBI 4 mm values of ≥400 or ≥500, which often indicate a plaque with a significant lipid core, associated with an increased risk of adverse cardiac events [96].

Translated clinically, high LCBI values (maxLCBI 4 mm ≥ 400–500) are associated with an elevated risk of MACE, including cardiac death, myocardial infarction, and target vessel revascularization. Increased values indicate vulnerable plaque features, such as thin fibrous caps, large lipid pools, and macrophage infiltration, while also predicting an increased risk of periprocedural myocardial infarction during PCI, likely due to distal embolization of lipid-rich material.

LCBI measurement is most valuable in patients undergoing coronary angiography or PCI, particularly those with acute coronary syndrome or stable angina pectoris and helps identify patients who may benefit from intensive medical therapies or lifestyle interventions. As a dynamic marker, it can be used to assess the response to treatments aimed at reducing lipid-rich plaque burden.

Clinical studies using near-infrared spectroscopy (NIRS) and IVUS have shown that adding PCSK9 inhibitors to statin therapy leads to a greater reduction in both LDL-C levels and the lipid core burden of coronary plaques. Specifically, PCSK9 inhibitors promote greater regression of the maxLCBI 4 mm compared to statin therapy alone (57.0 vs. 25.5; *p* = 0.010), with a greater decrease in plaque lipid content and increased plaque stability [97]. The degree of LCBI reduction correlates linearly with LDL-C lowering: the more LDL-C is reduced, the more the lipid core within plaques shrinks, leading to stabilization of vulnerable, rupture-prone plaques [98]. A lowered LCBI after PCSK9 inhibitor treatment means plaques are less likely to rupture, decreasing the risk of major adverse cardiovascular events (MACE) like myocardial infarction, stroke, and death [99]. The use of PCSK9 inhibitors is associated with increased fibrous cap thickness and regression of overall atheroma volume, both indicators of plaque stabilization and reduced risk. PCSK9 inhibitors can produce meaningful reductions in plaque lipid burden and promote stabilization within weeks to months, particularly important after ACS [98]. PCSK9 inhibitors are beneficial for patients with high LCBI, particularly those who fail to reach LDL-C goals with statins. Despite evidence of plaque stabilization and lipid reduction, the FITTER trial [100] did not demonstrate a measurable improvement in physiological endpoints (maxLCBI 4 mm, FFR) in non-culprit coronary lesions over short-term follow-up. However, this trial has some limitations such as the short intervention period (12 weeks), small sample size, and a high proportion of statin-naïve patients (75%). Longer-term and larger studies may be needed to clarify the benefit of PCSK9 inhibitors for non-culprit lesion stabilization and clinical outcome.

## 11. Evidence to Start Immediately After ACS with Dual- or Triple-Lowering Lipid Therapy

Recently, numerous articles have promoted, especially for patients at high or very high risk, the initiation of a more aggressive lipid-lowering therapy even immediately after the acute coronary event. Data from the SWEDEHEART (Swedish Web-System for Enhancement and Development of Evidence-Based Care in Heart Disease Evaluated According to Recommended Therapies) registry show that in patients with ACS, 75–80% of them do not reach the therapeutic LDL-C targets (<1.4 mmol/L; 55 mg/dL) if they are treated with statin monotherapy [101].

In a recent study by Kunak Mahajan et al. presented at ESC Congress 2024, it was demonstrated that triple therapy, comprising rosuvastatin 40 mg daily, ezetimibe 10 mg daily, and bempedoic acid 180 mg daily, effectively and swiftly achieves LDL-C targets following an ACS event. In this investigation, 70.8% of patients receiving rosuvastatin, ezetimibe, and bempedoic acid (REB) achieved the LDL-C target of <50 mg/dL within four weeks (as recommended by the Lipid Association of India), while 67.6% of patients on rosuvastatin and ezetimibe (RE) reached this target. Only 50% of patients on high-intensity statins alone achieved this target. This data indicate that combination therapies, particularly the triple REB therapy, were more effective in helping patients reach the stricter LDL-C goal of <50 mg/dL after an acute coronary syndrome event [39].

Another study aimed to determine whether combining ezetimibe with a high-intensity statin (ezetimibe 10 mg, rosuvastatin 10 mg) offers a more effective and safer alternative therapy compared to high-intensity statin monotherapy (20 mg rosuvastatin) for patients with coronary atherosclerotic heart disease. The proportion of patients achieving an LDL-C goal of 55 mg/dL in the third month was significantly higher in the combination therapy group (Group 2) at 60% compared to the high-dose rosuvastatin monotherapy group (Group 1) at 33% (*p* = 0.001). A decrease in high-sensitivity CRP was observed, accompanied by no increase in blood transaminases, indicating a positive safety profile and an anti-inflammatory effect [102]. 

A nationwide, population-based, retrospective cohort study found that combination therapy is associated with a statistically significant reduction in the risk of adverse cardiovascular outcomes (HR 0.85, 95% CI 0.78–0.92, *p* < 0.001) compared to monotherapy [103]. This study provides evidence that, for patients with acute coronary syndrome, a combination of moderate-dose statin and ezetimibe is more effective in reducing adverse cardiovascular events than high-intensity statin monotherapy, as observed in real-world clinical settings.

A subanalysis of the PL-ACS (Polish Registry of Acute Coronary Syndromes) registry indicates that initial combination lipid-lowering therapy is more effective than statin monotherapy in reducing all-cause mortality among patients with acute ACS. A statistically significant difference in mortality rates was observed between the groups throughout the follow-up period, consistently favoring the initial combination therapy. Specifically, at 1 year, the mortality rate was 3.5% for combination therapy compared to 5.9% for monotherapy (*p* = 0.041); at 2 years, the mortality rate was 4.3% for combination therapy versus 7.8% for monotherapy (*p* = 0.019); and at 3 years, the mortality rate was 5.5% for combination therapy compared to 10.2% for monotherapy (*p* = 0.024). Upfront combination therapy was associated with a significant reduction in all-cause mortality compared to statin monotherapy (OR, 0.526; 95% CI, 0.378–0.733) [38]. The most important practical implication of this study is the recommendation to move away from a stepwise approach to lipid-lowering therapy in patients with ACS. Instead of initiating statin monotherapy and then adding ezetimibe if needed, the study suggests that an upfront combination of statin and ezetimibe should be the preferred initial strategy. The paper highlights explicitly that this upfront combination approach is particularly beneficial and should be recommended for high-risk ACS patients. Clinicians should identify these patients and prioritize this intensive therapy from the outset.

According to an analysis of the SWEDEHEART registry involving 35,826 patients, the early initiation of ezetimibe in conjunction with high-intensity statin therapy following myocardial infarction (MI) significantly reduces the risk of major adverse cardiovascular events (MACE) and cardiovascular mortality compared to delayed initiation or no ezetimibe therapy. Patients who commenced ezetimibe treatment within 12 weeks post-MI exhibited a lower incidence of MACE at one year (1.79 per 100 patient-years) compared to those with delayed initiation (2.58) or no ezetimibe treatment (4.03). The study further underscores the additional benefit of ezetimibe beyond statin monotherapy, as evidenced by the fact that over 98% of patients were treated with high-intensity statins [101].

A multidisciplinary expert panel strongly endorses the integration of combination LLT as a first-line treatment strategy for patients with high and very high CV risk, moving away from the traditional stepwise approach that begins with statin monotherapy

The European observational SANTORINI study, with a 1-year follow-up of patients at high or very high cardiovascular risk across 14 European countries, demonstrated that the use of combination lipid-lowering therapy (LLT) is associated with significantly improved LDL cholesterol (LDL-C) management compared to monotherapy [104].

A multidisciplinary expert panel strongly advocates for the adoption of combination lipid-lowering therapy (LLT) as a first-line treatment strategy for patients with high and very high cardiovascular (CV) risk, thereby moving away from the traditional stepwise approach that commences with statin monotherapy [105]. The experts call for a paradigm shift from an exclusive focus on high-intensity statins to a more comprehensive concept of high-intensity lipid-lowering therapy, ideally employing upfront combination therapy. This strategy is intended to address the significant residual cardiovascular risk associated with inadequate lipid management under the stepwise model. Empirical data from real-world settings indicate a substantial discrepancy between guideline-recommended low-density lipoprotein cholesterol (LDL-C) targets and their actual attainment in clinical practice. Early combination therapy, which includes statins in conjunction with ezetimibe and newer agents such as bempedoic acid, increases the probability of achieving LDL-C goals more effectively. The integration of newer LLTs such as bempedoic acid into treatment regimens is recognized as an important advancement. These agents provide additional LDL-C lowering options, particularly for patients who cannot tolerate high-dose statins or require further LDL-C reduction beyond statin and ezetimibe therapy. The experts emphasize the need for a paradigm shift from focusing solely on high-intensity statins to a broader concept of high-intensity lipid-lowering therapy, preferably utilizing upfront combination therapy. This approach aims to mitigate the considerable residual CV risk observed with inadequate lipid management under the stepwise model.

### 11.1. Reasons to Move to Early Combination Therapy Instead of Stepwise Therapy in Dyslipidemia Treatment for ACS Patients

Firstly, early combination therapy (e.g., high-intensity statin plus ezetimibe) achieves lower LDL-C levels more rapidly than the traditional stepwise approach, which starts with statin monotherapy. This accelerated LDL-C reduction is crucial for ACS patients who are at very high cardiovascular risk and require swift lipid control to prevent recurrent events [106]. Furthermore, several studies have shown that early combination therapy reduces the risk of MACE, cardiovascular death, and all-cause mortality more effectively than delayed or stepwise intensification. Early addition of ezetimibe to statins after MI is associated with significant absolute and relative benefits in reducing adverse cardiovascular outcomes. Patients with ACS face 20% mortality risk in the first year; early combination therapy lowers all-cause mortality (OR 0.53, 95% CI 0.38–0.73) [101]. In addition, only 6.7% of high-risk patients reach LDL-C targets with statin monotherapy versus 31.7% with combination therapy [105]. Stepwise approaches delay intensification, leaving patients vulnerable during high-risk periods [106]. Upfront combination therapy leads to a higher proportion of patients reaching stringent LDL-C targets (e.g., <55 mg/dL or <1.4 mmol/L) compared to statin monotherapy. This is important since many ACS patients fail to achieve LDL-C goals with statins alone, and early combination therapy helps overcome this treatment gap. Stepwise therapy fails to intensify treatment in >80% of ASCVD patients within two years, perpetuating suboptimal LDL-C control [107]. The stepwise approach often results in delayed intensification of therapy, leaving many patients undertreated for extended periods. Early combination therapy simplifies treatment escalation, reducing clinical inertia and enabling more timely attainment of lipid targets.

Although some guidelines still recommend stepwise therapy, recent expert consensus [105,108] and guidelines (e.g., 2022 ACC EDCP, International Lipid Expert Panel) [109] increasingly support early or upfront combination therapy in very-high-risk patients, including those with ACS, to improve outcomes and reduce residual risk. Combination therapy, including fixed-dose combinations, may improve adherence by reducing pill burden and is safe and well-tolerated. Patients on combination therapy also exhibit lower rates of dose reduction or discontinuation compared to those on monotherapy, supporting sustained lipid control.

Early combination allows for the timely introduction of non-statin agents, such as ezetimibe and PCSK9 inhibitors, which have additive LDL-C lowering effects and improve plaque stabilization, thereby further reducing cardiovascular risk after an acute coronary syndrome (ACS).

### 11.2. Current RCTs on Lipid-Lowering Therapy in Post-ACS Patients

Randomized controlled trials have established a compelling evidence base for intensive lipid-lowering therapy in acute coronary syndrome (ACS) patients, demonstrating progressively greater cardiovascular benefits with more aggressive LDL-C reduction strategies. The foundational PROVE IT-TIMI 22 trial (n = 4162) first demonstrated the superiority of high-intensity atorvastatin 80 mg over moderate-intensity pravastatin 40 mg, achieving 51% versus 22% LDL-C reduction and a 16% relative risk reduction in composite cardiovascular endpoints (HR 0.84, *p* = 0.005) [24]. Building on this foundation, the IMPROVE-IT trial (n = 18,144) showed that adding ezetimibe to simvastatin provided incremental benefit, with an additional 23% LDL-C reduction to <60 mg/dL translating to a 6% relative risk reduction in cardiovascular death, MI, and stroke (HR 0.94, *p* = 0.016) [110]. The paradigm of “lower is better” was further validated by PCSK9 inhibitor trials: ODYSSEY OUTCOMES (n = 18,924) demonstrated that alirocumab plus statin achieved 55% LDL-C reduction (to 53 mg/dL) with a 15% relative risk reduction in MACE (HR 0.85, *p* = 0.0003) [47], while the FOURIER trial’s post-ACS subgroup showed similar benefits with evolocumab achieving 59–66% LDL-C reduction (to 30 mg/dL) and 15% MACE reduction (HR 0.85, *p* < 0.001) [111]. Emerging therapies include bempedoic acid (CLEAR Outcomes trial), which achieved 21% LDL-C reduction with promising cardiovascular benefits under investigation [112] and decision support systems (ZODIAC trial, n = 1250 ACS patients) that improved LDL-C target achievement but did not significantly impact cardiovascular endpoints. Table 1 provides detailed comparative data from these landmark trials, including specific patient populations, interventions, outcomes, and achieved LDL-C reductions for each study (Table 1).

### 11.3. Insights from Large Real-World Databases

In the nationwide SWEDEHEART registry, only 17.1% of the 25,466 MI patients reached the newly set LDL-C target (<1.4mmol/L), meaning that over 80% would be eligible for intensification and combination lipid-lowering therapy. Simulations suggest that up to 92% could achieve targets if combination therapies (statins, ezetimibe, PCSK9 inhibitors) were implemented more broadly. Most patients receive statin monotherapy after ACS; however, only a minority achieve LDL-C goals—typically 18–20% as per registry and clinical trial data. SWEDEHEART studies highlight that earlier initiation of ezetimibe and other combinations post-MI is associated with improved long-term prognosis, with up to 70% reduction in major cardiovascular events and all-cause mortality over a median follow-up of 3.7 years when LDL-C reduction is rapid (e.g., 2.0 mmol/L drop within 4–6 weeks) [112]. Participation in structured cardiac rehabilitation programs further increases LDL-C target achievement rates in post-ACS patients. After such programs, 26% achieve both LDL-C reduction and target level, with up to 76% achieving one or both goals [112].

SWEDEHEART registry data demonstrate that while high-intensity and combination lipid-lowering therapy post-ACS can enable most patients to achieve ambitious ESC/EAS LDL-C targets, real-world implementation lags, primarily due to delayed initiation and suboptimal use of combination therapies. Early, aggressive, and sustained lipid-lowering regimens (statin plus ezetimibe, then expanders like PCSK9 inhibitors or bempedoic acid as needed) are key to improving outcomes and reducing future cardiovascular events in these high-risk patients [101].

Observational databases are subject to selection bias, missing data, treatment heterogeneity, and incomplete adherence reporting. Despite existing guidelines, achieving optimal LDL-C levels remains suboptimal. System-level challenges, such as reimbursement policies and prescription practices, along with the underutilization of potent therapies, are common.

When comparing randomized controlled trials (RCTs) to the SWEDEHEART registry data regarding lipid-lowering therapy after acute coronary syndrome (ACS), several important differences emerge in design, patient populations, interventions, and outcomes.

RCTs are designed to test the efficacy and safety of specific lipid-lowering regimens in well-defined groups under controlled conditions. They typically enroll selected patients with strict inclusion and exclusion criteria, ensuring uniform baseline characteristics and monitoring adherence closely. As a result, patients in RCTs tend to be younger, healthier, and free of major comorbidities, creating a scenario where the benefits and side effects of therapies can be measured precisely. Treatment protocols are standardized and closely followed, often including robust adherence measures and frequent follow-ups, which leads to higher rates of LDL-C goal achievement than in routine practice settings.

In contrast, the SWEDEHEART registry captures real-world data from a nationwide cohort of unselected ACS patients treated during daily clinical practice. This means the registry reflects the heterogeneity of actual patients—including older individuals, those with multiple comorbidities, and varying degrees of risk. The registry data illustrate how lipid-lowering therapies are implemented outside of clinical trials, demonstrating lower rates of LDL-C target achievement compared to RCTs. These discrepancies stem largely from less frequent use of combination regimens, differences in patient adherence, delayed therapy initiation, and under-prescribing of additional lipid-lowering agents. Follow-up intervals are longer and less structured, which reflects challenges in routine care, and outcomes are influenced by a broader spectrum of patient factors that are not addressed in RCTs.

RCT outcomes typically represent the best-case scenario for therapy efficacy, while the SWEDEHEART registry provides a realistic view of effectiveness and implementation gaps in managing post-ACS patients. Most notably, SWEDEHEART data show that guideline goals are reached by far fewer patients, with many eligible for further intensification, underlining the need for earlier and more aggressive lipid-lowering strategies in routine care compared to what is typically observed in RCTs.

### 11.4. The Future of Lipid-Lowering Therapy: Current Gaps and Further Studies

Future lipid-lowering therapy is distinguished by the advent of novel agents and mechanisms evidenced in recent and ongoing clinical trials such as CLEAR Outcomes, VICTORION-2 Prevent, and the ORION program. The CLEAR Outcomes trial establishes bempedoic acid as an effective tool for patients intolerant to statins, demonstrating significant reductions in LDL-C and associated cardiovascular events in populations both with and without established atherosclerotic cardiovascular disease, without an adverse impact on glycemic control and with benefits observed even among diabetes cohorts [111,113]. This large-scale, randomized, placebo-controlled trial enrolled a highly representative patient population. It confirmed the incremental value of bempedoic acid for primary and secondary prevention when statins cannot be used [112].

Patients with ACS were not recruited explicitly as a distinct group in the CLEAR Outcomes trial; instead, eligibility was based on having a history of ASCVD—which includes prior myocardial infarction, unstable angina, or coronary revascularization—or being at high risk of cardiovascular events along with statin intolerance and elevated LDL-C. The majority of trial participants were in the secondary prevention category (about 70%), meaning a substantial proportion suffered previous cardiovascular events compatible with ACS, such as myocardial infarction or unstable angina requiring intervention. Still, enrollment was not restricted to new-onset ACS cases nor explicitly stratified by ACS status. Patients with a prior history of ACS were therefore included under the broader ASCVD criteria. However, the trial was not designed as an acute-phase ACS study and did not report outcomes exclusively for ACS patients. For focused research in the ACS population, a separate trial, CLEAR ACS, is ongoing.

Recent randomized trials, such as ES-BempedACS, have evaluated the efficacy and safety of adding bempedoic acid to high-potency statin plus ezetimibe therapy in the immediate post-ACS setting. In this multicenter study, post-ACS patients randomized within 72 h to triple oral therapy (statin, ezetimibe, bempedoic acid) versus dual therapy (statin, ezetimibe) showed similar proportions in achieving targeted LDL-C levels (<55 mg/dL) at 8 weeks (59.4% vs. 53.1%, *p* = 0.375), with comparable absolute LDL-C reductions from baseline in both groups. Triglyceride and non-HDL-C levels were likewise similar, and most frequent adverse events, such as hyperuricemia, occurred at similar rates, supporting safety in the acute setting [63].

The VICTORION clinical program, notably the VICTORION-2 Prevent study, and associated trials such as V-MONO, have provided pivotal insights into inclisiran administered twice yearly. The siRNA delivers sustained and potent LDL-C reductions superior to both ezetimibe and placebo, with robust safety and tolerability confirmed across diverse populations, including those not receiving baseline lipid-lowering therapy or at risk for diabetes [114]. VICTORION integrates randomized controlled and real-world evidence, aiming to position inclisiran for broad utility in high-risk and statin-intolerant patients and anticipates outcome-driven confirmation for cardiovascular event reduction.

The VICTORION-INCEPTION (NCT04873934) trial was the first prospective, randomized clinical study to assess the LDL-C-lowering effects and safety of inclisiran in patients discharged after recent ACS (within 5 weeks before screening). This trial compared inclisiran plus usual care to usual care alone, including statins and other lipid-lowering therapies, and demonstrated rapid, meaningful, and sustained lowering of LDL-C—with a 45.6% reduction in the inclisiran group versus a 1.4% decrease in standard care at 5 months. Safety was favorable, with mild-to-moderate injection site reactions as the most common adverse event.

The ORION program reinforces these findings at scale, highlighting the persistence of LDL cholesterol lowering with inclisiran over multi-year follow-up and broad patient applicability, including those with established cardiovascular disease, familial hypercholesterolemia, and baseline high LDL-C, and further targets outcome evidence through ongoing trials such as ORION-4. Collectively, these programs signal a transition in lipid management, moving beyond conventional therapies toward durable, infrequent-dose, and mechanism-diverse approaches, offering promise for enhanced efficacy, adherence, and patient outcomes across global populations at risk for cardiovascular disease.

Earlier pivotal trials on inclisiran—such as ORION-9, ORION-10, and ORION-11—enrolled broad atherosclerotic cardiovascular disease populations, sometimes including patients with a history of ACS, but were not designed as ACS-specific studies. Dedicated evidence for inclisiran’s impact post-ACS is therefore emerging from these newer, ACS-focused trials.

Clinical trials investigating inclisiran in ACS are ongoing. However, published evidence to date has primarily addressed its use in broader ASCVD populations rather than enrolling ACS patients as an exclusive cohort. The most notable ongoing trial specifically in ACS is a randomized study titled “Evaluation of Efficacy and Safety of Early Inclisiran Therapy in Patients with Acute Coronary Syndromes” (ClinicalTrials.gov NCT07102628) designed to assess the effects of inclisiran in individuals with ACS. This trial is actively recruiting and aims to address early therapy post-ACS. Early real-world implementation studies (e.g., VICTORION-INCEPTION) also target high-risk patients in the first year after ACS. However, complete results are not yet available, and published phase 3 trials (ORION program) have not stratified ACS status at enrollment but included patients with established ASCVD, which may have encompassed recent ACS cases. Thus, while dedicated ACS-specific trials are ongoing, inclisiran’s role post-ACS remains under clinical investigation, with initial data anticipated to clarify efficacy, safety, and cardiovascular outcomes in this high-risk population (Table 2).

Current gaps in knowledge about lipid-lowering therapy after acute coronary syndrome largely stem from differences between controlled clinical trial results and real-world registry findings. While randomized controlled trials have demonstrated clear outcome benefits of intensive and combination lipid-lowering strategies, registry studies such as SWEDEHEART consistently show that most patients do not reach guideline-recommended LDL-C targets—even with maximal statin therapy and despite improvements over time. This gap reflects diverse patient populations, underuse of combination therapies, suboptimal follow-up, and barriers in routine care such as cost, adherence, and practitioner knowledge [115].

Several knowledge gaps remain, such as the best timing and intensity of initiation for combination regimens (especially in the immediate post-ACS phase) or the effect of very low LDL-C levels on long-term safety and outcomes in broader, unselected populations, which are the consequences and potential benefits of early aggressive “strike early, strike strong” approaches in varied real-world settings. Furthermore, strategies to overcome system-level implementation challenges must be developed to ensure prompt follow-up, identify candidates for therapy escalation, and optimize adherence in clinical practice.

The effectiveness and safety of newer agents (such as PCSK9 inhibitors and inclisiran) started immediately after an ACS must be explored, as most outcome trials enrolled patients later after the index event.

To address these unresolved issues, several ongoing and recently launched trials hold promise for providing more robust evidence.

Ongoing trials (Table 3) such as the Amundsen study possess high potential for influencing future lipid therapy guidelines by addressing key questions about timing, intensity, and choice of agents for LDL-cholesterol reduction after acute coronary syndrome. Amundsen is the first randomized study to evaluate immediate initiation of a potent lipid-lowering agent (evolocumab) before percutaneous coronary intervention in patients with STEMI or NSTEMI, measuring not just lipid targets but also hard clinical outcomes like cardiovascular mortality and ischemic events after 12 months [116].

Assuming the Amundsen trial demonstrates that very early, intensive combination therapy (adding PCSK9 inhibitors up front to statins and ezetimibe) leads to more rapid attainment of guideline LDL targets and improved cardiovascular outcomes, this will provide a stronger level of evidence favoring earlier and more aggressive therapy—shifting the paradigm from the traditional stepwise approach. Such results could lead to future guidelines recommending upfront combination therapy for very-high-risk ACS patients, rather than waiting to escalate based on initial response. Guidelines may also set new standards for LDL-C targets and reinforce the concept of “strike early, strike strong” routinely in ACS care, with more emphasis on rapid and profound lipid reductions in the immediate aftermath of an event.

Additionally, if we assume that safety and long-term outcome data are favorable, guideline committees may endorse immediate use of agents like PCSK9 inhibitors and inclisiran not just for patients who fail standard therapy, but as part of initial therapy post-ACS, especially for those with very high LDL levels or extensive atherosclerotic burden. The Amundsen and other ongoing studies will clarify how best to implement these therapies in routine practice, optimize timing and sequencing, and overcome current gaps seen in real-world registry data, potentially making early combination therapy standard practice worldwide.

Ongoing trials like VICTORION-INCEPTION and the ORION series are set to influence future lipid therapy guidelines in ACS by generating robust evidence on the early use and real-world effectiveness of novel agents such as inclisiran.

Findings from the VICTORION program, especially the VICTORION-INCEPTION trial, are set to have major implications for future lipid-lowering therapy strategies after acute coronary syndrome (ACS). These results show that adding inclisiran to standard care produces rapid and sustained LDL-cholesterol reduction—nearly double of what is achieved with statin-based therapy alone—with a favorable safety profile. This finding is crucial, as more patients reach LDL-C targets within the first year after ACS [117]. This evidence points toward a new strategy in lipid management that emphasizes early, aggressive combination therapy rather than the traditional stepwise escalation. With inclisiran’s biannual dosing schedule, strategies can prioritize prompt, durable LDL-C lowering and improved adherence, making it more feasible in real-world clinical practice. Guidelines may evolve to recommend systematic use of agents like inclisiran for high-risk ACS patients, either at discharge or soon after, especially for those not at LDL-C goal on maximal oral therapies.

In effect, VICTORION findings support a “strike early, strike strong” approach—aiming for rapid and profound lipid reduction in all eligible ACS patients. This could lead to widespread adoption of combination regimens, expanded eligibility for novel therapies, and stronger systems to ensure patients receive timely and effective secondary prevention. The shift may bring lipid management in routine care closer to results seen in clinical trials, helping more patients reach guideline targets and reducing the risk of future cardiovascular events.

The VICTORION clinical program and the ORION trials are designed to address broader questions about the safety, practicality, and efficacy of inclisiran in large and diverse patient populations, including those underrepresented in earlier lipid-lowering trials. For example, the ongoing ORION-4 trial assesses the impact of inclisiran on major adverse cardiovascular events (MACE) in 15,000 participants with established ASCVD, echoing the endpoints most relevant to future guideline recommendations.

Inclisiran, studied in the ORION-9, ORION-10, and ORION-11 trials, delivers sustained LDL-cholesterol reductions of around 48–52% over 18 months in high-risk populations. Most participants achieved guideline LDL-C targets even when already on maximally tolerated statins. The therapy’s long-term efficacy is supported by extension studies showing consistent LDL-C reductions over several years with a twice-yearly dosing schedule. Inclisiran also lowers total cholesterol, non-HDL cholesterol, apolipoprotein B, and triglycerides, benefiting both primary and secondary prevention patients. Its safety profile is favorable, marked mainly by mild injection site reactions and no major organ toxicity. The durable effects and simple dosing have the potential to improve adherence compared to daily oral medications, making it practical for routine clinical care. Having a much larger proportion of high-risk patients achieve guideline LDL-C goals could reduce cardiovascular events and help bridge the gap between clinical trial results and real-world outcomes. These findings position inclisiran as a valuable addition to lipid-lowering strategies and support the evolution of guidelines toward earlier and more aggressive LDL-C reduction in high-risk patients.

The collective insights derived from these trials are anticipated to support the initiation of earlier and more aggressive LDL-C reduction following ACS, including the immediate incorporation of novel agents alongside statins and ezetimibe as well as provide evidence for the development of new treatment algorithms within guidelines, advocating for combination therapies shortly after hospitalization for ACS, rather than reserving additional agents for patients who later fail to achieve target levels.

If hard endpoint data from these large outcome studies confirm reductions in recurrent events and mortality, future lipid guidelines for ACS patients will likely recommend prompt initiation of combination therapy—including agents like inclisiran—not just for those who fail statins, but as routine practice for all very-high-risk individuals following acute coronary events.

## 12. Conclusions

Dyslipidemia is a critical modifiable risk factor in patients with acute coronary syndrome (ACS), and its optimal management is essential for reducing the risk of recurrent cardiovascular events. Current guidelines strongly recommend early and intensive lipid-lowering therapy to achieve ambitious LDL-C targets—preferably less than 1.4 mmol/L (<55 mg/dL) and at least a 50% reduction from baseline, with even lower targets for patients experiencing recurrent events. High-intensity statins (atorvastatin or rosuvastatin) remain the cornerstone of therapy, but a growing body of evidence supports the early addition of ezetimibe and, when necessary, PCSK9 inhibitors to maximize LDL-C reduction and improve clinical outcomes. Recent data demonstrate that combination therapy, particularly the upfront use of statins with ezetimibe or even triple therapy (statin, ezetimibe, and bempedoic acid), leads to faster and more robust LDL-C lowering and significantly reduces the risk of major adverse cardiovascular events and mortality compared to a traditional stepwise approach. This strategy also helps overcome clinical inertia and ensures that a greater proportion of high-risk patients achieve guideline-recommended lipid targets. Beyond LDL-C, assessment of residual risk should include evaluation of HDL-C, triglycerides, Lp(a), and remnant cholesterol, as these markers may identify patients who could benefit from additional targeted therapies. Emerging agents such as ANGPTL3 and apoC-III inhibitors, as well as novel therapies like inclisiran and icosapent ethyl, offer promising avenues for further reducing cardiovascular risk in patients with persistent dyslipidemia after ACS.

In summary, the management of dyslipidemia in ACS requires a proactive, individualized, and often combination-based approach, integrating both established and emerging therapies to address the full spectrum of lipid-related risk factors and to optimize long-term cardiovascular outcomes.

## Figures and Tables

**Table 1:** Randomized controlled trials of lipid-lowering therapies in acute coronary syndromes.

Study Name	No. of ACS Patients	Comparative Therapy	Outcome	Result	LDL-C Reduction Achieved	Criticism
PROVE IT-TIMI 22	4162	Atorvastatin 80 mg vs. Pravastatin 40 mg	Composite CV endpoint	High-intensity statin reduced event risk (HR, 0.84; *p* = 0.005)	Atorvastatin: 51%; Pravastatin: 22%	Modest absolute risk reduction; short follow-up
IMPROVE-IT	18,144	Simvastatin 40 mg + Ezetimibe vs. Simvastatin 40 mg	CV death, MI, stroke	Combo reduced events (HR, 0.94; *p* = 0.016)	Ezetimibe + statin: ~23% additional reduction (to LDL-C < 60 mg/dL)	Small absolute risk reduction; most patients on background statin
ODYSSEY OUTCOMES	18,924	Alirocumab + statin vs. statin alone	MACE (CV death, MI, stroke)	Alirocumab reduced events (HR, 0.85; *p* = 0.0003)	Alirocumab + statin: ~55% (to LDL-C 53 mg/dL from 92 mg/dL)	Excluded patients in acute phase; high cost of PCSK9 inhibitors
FOURIER	Subgroup: post-ACS not primary, whole trial 27,564	Evolocumab + statin vs. statin alone	MACE	Evolocumab reduced events (HR, 0.85; *p* < 0.001)	Evolocumab + statin: ~59–66% (to LDL-C 30 mg/dL from 92 mg/dL)	Did not include patients during acute ACS; high cost, unclear long-term safety
CLEAR Outcomes	Unclear for ACS; trial included high-risk patients, not strictly ACS	Bempedoic acid + statin vs. statin alone	CV events, safety	Bempedoic acid further lowers LDL-C; CV benefits under investigation	Bempedoic acid: 21% reduction	Full outcome data not yet available; limited long-term safety data
ZODIAC	Subgroup: ACS admission, 1250	DSS-guided vs. usual care in lipid lowering	LDL-C target achievement	DSS improved early achievement of targets (54.7% vs. 48.7%)	Not primarily reported	Did not show significant difference in primary endpoint; possible Hawthorne effect

**Table 2:** Current studies focusing on inclisiran as lipid-lowering therapy.

Trial Name	N Patients (Total)	ACS/ASCVD Patients	Comparative Therapies	Primary Outcomes	LDL-C Reduction (%)	Results	Limitations
ORION-1	497	Majority ASCVD; ACS included (not exclusively)	Inclisiran vs. placebo (±statins)	% change in LDL-C over 180 days	~50% (Day 180)	Durable LDL-C fall; consistent safety	Small Phase II, short duration
ORION-3	382 (extension of ORION-1)	ACS/ASCVD enrichment	Inclisiran-only vs. switching (evolocumab→inclisiran)	LDL-C change over 4 years, safety	~44% mean over 4 years	Sustained LDL-C reduction, 62–77% PCSK9 drop	Open-label, selection bias
ORION-10	1561	ASCVD (including ACS)	Inclisiran vs. placebo on top of statins	% LDL-C drop at month 17	52% at 17 months	75% achieved LDL-C < 55 mg/dL; consistent safety	Surrogate endpoint focus, event data pending
ORION-11	1617	ASCVD (including ACS)	Same as above	Same	≥50% at 18 months	75% attained LDL-C < 55 mg/dL	Similar limitations as above
ORION-4 (CVOT, ongoing)	~15,000 (target)	Atherosclerotic CVD, including ACS	Inclisiran vs. placebo on standard therapy	Major CV events (MACE)	Pending	Outcome pending	Double-blind outcomes; results awaited
ORION-8 (extension)	3274	ASCVD/high-risk mix	OLE inclisiran (no comparator)	LDL-C goal attainment, safety	~49% over 3 years	78% achieved LDL-C goals (ASCVD)	Open-label, no control group

**Table 3:** Ongoing trials of lipid-lowering therapy in acute coronary syndrome.

Trial Name	No. of Patients (Target)	Cohorts	Comparative Therapy	Outcomes (Primary)	LDL-C Reduction (Expected)	Criticisms/Limitations
EVOLVE-MI (NCT05284747)	4000	All post-AMI/ACS patients	Evolocumab + standard LLT vs. standard LLT	MACE (CV death, MI, etc.)	~60% reduction (PCSK9i + statin)	Not yet reporting outcomes; cost and access
AMUNDSEN (NCT04951856)	1666	All ACS (first dose pre-PCI)	Early evolocumab vs. standard therapy	LDL-C reduction, tertiary MACE	~65% reduction (similar to EVOPACS)	Focus primarily on LDL-C as endpoint
VICTORION-INCEPTION (NCT04873934)	~380	Recent ACS (within 5 weeks)	Inclisiran + statin vs. standard therapy	1-year LDL-C reduction, lipid targets	~50% reduction (expected for inclisiran)	Outcomes mainly surrogate/lipid-based
PACMAN-AMI [recently completed]	300	PCI for acute MI	Alirocumab vs. placebo on statins	IVUS plaque regression, not events	85% achieved LDL-C < 55 mg/dL in 1yr	Imaging endpoints, not clinical events
EPIC-STEMI	68	STEMI	Alirocumab + statin vs. placebo	% LDL-C reduction at 6 weeks	72.9% (alirocumab) vs. 48.1% (placebo)	Small sample, short duration
HUYGENS	161	MI patients	Evolocumab vs. placebo (statin-based)	Plaque stabilization, LDL-C	LDL-C reduced to 28.1 mg/dL (~80% reduction)	Mechanistic, not powered for events

## Data Availability

No new data were created or analyzed in this study. Data sharing is not applicable to this article.

## Abbreviations

The following abbreviations are used in this manuscript:

ACC	American College of Cardiology
ACS	Acute Coronary Syndrome
AHA	American Heart Association
ASCVD	Atherosclerotic Cardiovascular Disease
BMI	Body Mass Index
BP	Blood Pressure
CAD	Coronary Artery Disease
CI	Confidence Interval
CRP	C-Reactive Protein
CV	Cardiovascular
CVD	Cardiovascular Disease
DASH	Dietary Approaches to Stop Hypertension
DM	Diabetes Mellitus
ESC	European Society of Cardiology
EVOPACS	Evolocumab for Early Reduction in LDL cholesterol levels in Patients With Acute Coronary Syndromes
FPP	Farnesyl Pyrophosphate
HDL	High-Density Lipoprotein
HDL-C	High-Density Lipoprotein Cholesterol
HMG-CoA	Hydroxymethylglutaryl-Coenzyme A
HR	Hazard Ratio
IPE	Icosapent Ethyl
LCBI	Lipid Core Burden Index
LDL	Low-Density Lipoprotein
LDL-C	Low-Density Lipoprotein Cholesterol
LLT	Lipid-Lowering Therapy
MACE	Major Adverse Cardiovascular Events
MI	Myocardial Infarction
NIRS	Near-Infrared Spectroscopy
NSTEMI	Non-ST-Elevation Myocardial Infarction
OCT	Optical Coherence Tomography
OR	Odds Ratio
PACT	Pravastatin in Acute Coronary Treatment
PCI	Percutaneous Coronary Intervention
PCSK9	Proprotein Convertase Subtilisin/Kexin Type 9
RCT	Randomized Controlled Trial
RR	Relative Risk
STEMI	ST-Elevation Myocardial Infarction
SWEDEHEART	Swedish Web-system for Enhancement and Development of Evidence-based care in Heart disease Evaluated According to Recommended Therapies
TG	Triglycerides
UA	Unstable Angina
VLDL	Very-Low-Density Lipoprotein
hs-CRP	High-Sensitivity C-Reactive Protein
siRNA	Small Interfering Ribonucleic Acid
